# The Infection of Phthisis

**Published:** 1893-12-30

**Authors:** 


### The Infection of Phthisis.

A wise and important step has been taken by Dr. Herman Biggs, of New York. He has officially declared the contagiousness of consumption, and recommended the Board of Health to adopt measures for the prevention of the spread of the disease. Dr. Biggs suggests that the public should be educated by means of circulars and other publications to realise the importance of preventive measures. Further, it is proposed that persons suffering from chest affections, or their medical men, shall be required to report to the Board of Health where the disease exists, that proper instruction may be given in respect to disinfection. It is considered undesirable that pulmonary complaints should be excluded from the general wards of hospitals, and that distinct hospitals for consumption should be encouraged. If these measures are regarded as of such necessity in America by health authorities, how much more strenuously should they commend themselves for adoption in our metropolis. Here pulmonary complaints are rife, and it is commonly said that something to lessen the chances of infection should be done, but no scheme, such as Dr. Biggs', has yet been formulated amongst us, and considering that its success depends much upon education, no time should be lost in commencing a method of necessarily slow attainment.

